# A standardised communication tool reduces radiation exposure associated with intraoperative fluoroscopy

**DOI:** 10.1007/s11845-023-03442-2

**Published:** 2023-07-14

**Authors:** Timothy McAleese, Alexander Price, Anthony G. Ryan, Fiachra E. Rowan

**Affiliations:** 1 Department of Trauma and Orthopaedic Surgery, University Hospital Waterford, Waterford, X91ER8E Ireland; 2 Department of Radiology, University Hospital Waterford, Waterford, X91ER8E Ireland

**Keywords:** Communication tool, Intraoperative fluoroscopy, Orthopaedic surgery, Radiation exposure, Standardised commands

## Abstract

**Background:**

The widespread use of intraoperative fluoroscopy in orthopaedic procedures has revolutionised surgical practice. However, there are risks associated with using ionising radiation. Efforts to reduce radiation exposure include low-dose imaging protocols and lead protective equipment. Current communication during fluoroscopic procedures can be inefficient and lead to excessive radiation exposure for patients and staff.

**Aims:**

This study aims to implement a communication tool with standardised commands to reduce radiation exposure in an Irish orthopaedic department.

**Methods:**

Radiation exposure was evaluated using dose-area product (DAP) measured in uGy/m2. A control group was recorded before implementing the communication tool. Training sessions were conducted and posters of the standardised commands were displayed. Feedback was collected from surgeons and radiographers via surveys. Statistical analysis was performed to compare pre- and post-intervention groups.

**Results:**

A total of 673 surgical cases were included over 6 months. The post-intervention group showed a mean reduction in radiation exposure from 59.8 to 36.4 uGy/m2 (*p* < 0.011). Subset analyses revealed reduced radiation exposure for ORIF of the distal radius, ankle, humerus, and phalanges. Surgeons and radiographers recognised the need for improved communication and expressed willingness to learn the new tool.

**Conclusions:**

Implementation of a standardised communication tool effectively reduced patient and staff radiation exposure. It was also believed to have a positive effect on theatre staff morale. Incorporating a universal language tool into training programmes could be beneficial. Surgeons and radiographers provided several suggestions to improve the effectiveness and implementation of this tool into other units.

## Background

The use of intraoperative fluoroscopy during orthopaedic procedures has revolutionised surgical practice since its widespread adoption in the 1980s. However, using ionising radiation comes with inherent risks to both patients and medical staff. Excessive exposure can lead to numerous health problems, including malignancy, dermatitis, cataracts, and disorders of the thyroid [1, 2]. This has provoked efforts aimed at reducing radiation exposure such as low-dose imaging protocols, collimation, lead protective equipment and monitoring staff dosimeters. The fundamental concept guiding the utilisation and safe practice of medical radiation is to use doses “as low as reasonably achievable” (ALARA) [3].

 Radiation exposure could also be reduced by improving communication between surgeons and radiographers while using intraoperative C-arm fluoroscopy. Ambiguous instructions can lead to repeated x-rays, time inefficiency, and staff frustration. Establishing standardised commands has been proven to be effective in reducing radiation exposure and maximising time efficiency in simulated settings, although more research examining the clinical impact of this is required [4, 5]. Other survey-based studies have revealed that both surgeons and radiographers recognise that a universal language would have significant benefits and acknowledge the need for formal training in this area [6, 7].

This research paper analyses the implementation of a reproducible, standardised communication tool in an Irish orthopaedic department. Our primary objective was to reduce radiation exposure to patients and staff by improving theatre staff communication. Secondarily, we explored how to develop this tool further and streamline its introduction into other orthopaedic departments by collecting feedback from surgeons and radiographers.

## Methods

**Fig. 1:**
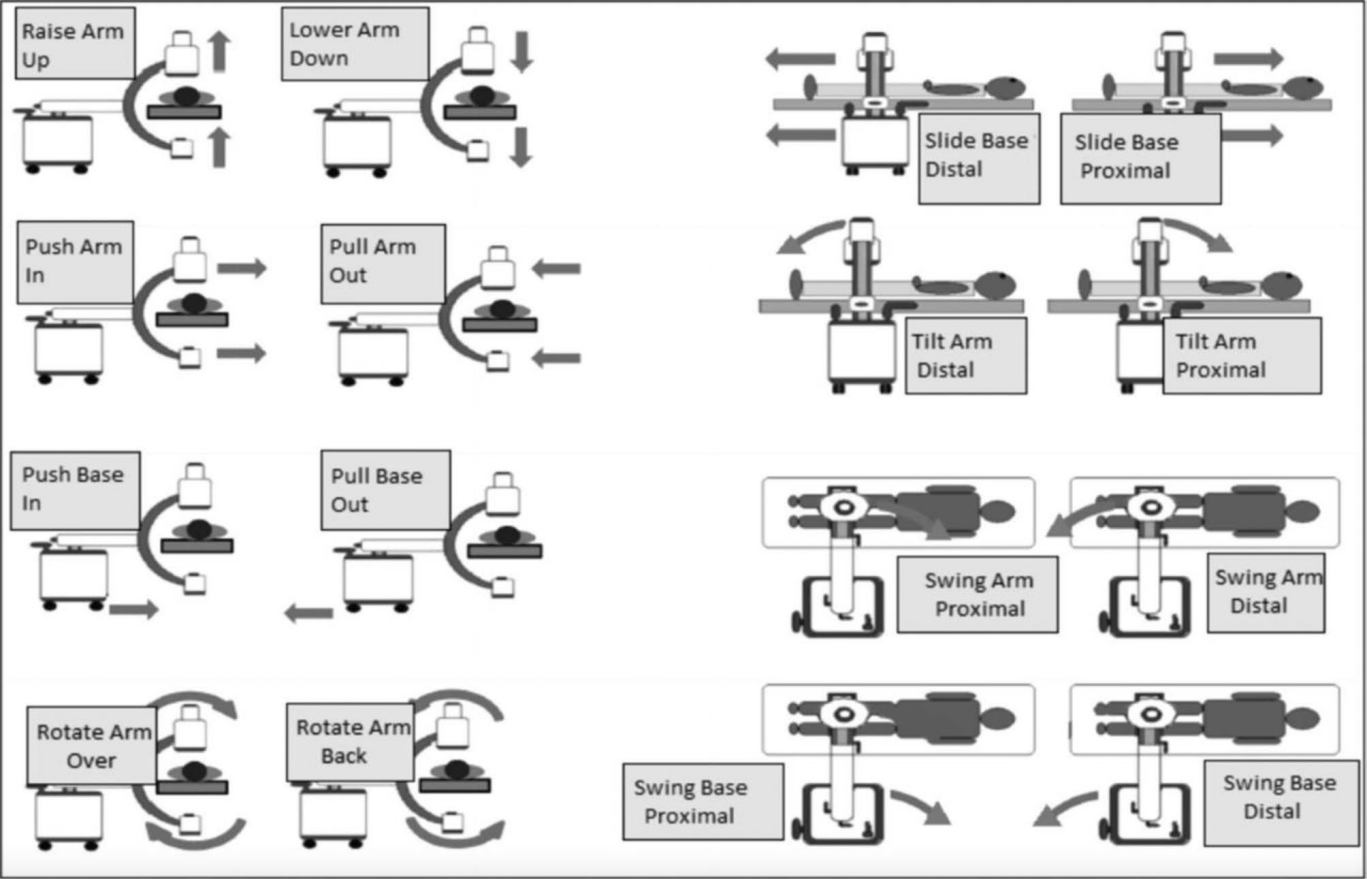
The standardised nomenclature assigned to different C-arm movements designed by Stirton et al. [5]. Theatre staff were trained to use this language, and posters were displayed

This study took place in University Hospital Waterford, a trauma unit in Ireland, over a period of 6 months. To create a control group, all orthopaedic procedures that required intraoperative C-arm fluoroscopy were recorded over a 2-month period between July and August 2022, before implementation of the standardised communication tool. Following this, two training sessions on how to use the communication tool were held, one for surgeons and one for radiographers. Posters of the pre-defined commands were then displayed in the orthopaedic theatres (Fig. 1). The communication tool implemented was proposed by Stirton et al. and demonstrates 16 distinct movements of the C-arm or its base using intuitive and clear commands [6].

Over the next 2 months staff familiarised themselves with the commands and a survey was conducted to gather feedback from the surgeons and radiographers who had used the preliminary tool. The survey assessed staff attitudes towards current intraoperative C-arm use and evaluated how the tool could be optimised. This was performed using a 5-point Likert scale. We also received feedback on how to streamline the tool’s implementation into practice. Post-intervention data was collected from all trauma cases requiring C-arm fluoroscopy over another 2-month period between November and December 2022.

Radiation absorption per case was evaluated using the dose-area product (DAP) measured in uGy/m2. This was available at the end of each case in the radiation summary provided by the “National Integrated Medical Imaging System” (NIMIS, McKesson, Irving, TX, USA) radiology system. A comparison was made between the groups before and after the intervention. We performed a subgroup analysis of the most common orthopaedic cases in our cohort to improve the accuracy of our results. These cases were distal radius open reduction internal fixation (ORIF), ankle ORIF, proximal femur cephalomedullary nailing (CM nailing), humerus ORIF, and finger phalanx manipulation ± wiring.

Univariate analysis was used for descriptive statistics. Continuous variables are displayed as mean (± standard deviation), whereas categorical variables are displayed as number and percentage. The Mann–Whitney *U* test was used to compare proportions of unpaired, continuous, parametric data. The threshold for statistical significance was set at a *p* value < 0.05. Data analysis and graphical presentation were performed using SPSS version 29.

## Results

There were a total of 673 surgical cases included in the analysis over the 6-month period, 366 in the pre-intervention group and 307 in the post-intervention group. In our subset analysis, there were 146 patients who underwent distal radius ORIF (77 pre-intervention vs 69 post-intervention), 85 who underwent Ankle ORIF (44 pre- vs 41 post-intervention), and 79 proximal femur CM Nails (36 pre- vs 43 post-intervention). We also analysed 55 finger phalanx fractures (30 pre- vs 25 post-intervention) and 35 humerus ORIF (19 pre- vs 16 post-intervention).

Overall, there was a mean reduction in radiation exposure from 59.8 uGy/m2 (SD 130.4 uGy/m2) down to 36.4 (SD 81.6 uGy/m2) in the post-intervention group, *p* < 0.011 (Table 1). In our subset analysis, we found the intervention reduced radiation exposure in all groups except the proximal femur CM nailing group. In the distal radius ORIF group, there was a 15.3% decrease in radiation exposure from 3.7 to 3.1 uGy/m2, *p* = 0.62. In the ankle ORIF group, there was a 16.4% reduction in radiation from 10.3 to 8.6 uGy/m2, *p* = 0.975. In the humerus ORIF group, there was a 16.9% reduction from 71.8 to 59.7 uGy/m2, *p* = 0.693. Radiation exposure in the phalanx group reduced by 3.2% from 2.8 to 2.7 uGy/m2, *p* = 0.761. There was an increase of 32.6% in the dose-area product experienced by the patients in the proximal femur CM nailing group from 205.5 to 273.9 uGy/m2, *p* < 0.046 (Table 1).

**Table 1:** Comparison of the mean dose-area product (DAP) between the pre- and post-intervention groups, measured in uGy/m2

	**Procedures**	**Pre-intervention** (uGy/m2)	**Post-intervention** (uGy/m2)	**% decrease**	***p* value**
**Total**	673	59.8	36.4	− 39.1%	*p* < 0.011
Distal radius ORIF	146	3.7	3.1	− 15.3%	*p* = 0.62
Ankle ORIF	84	10.3	8.6	− 16.4%	*p* = 0.975
Proximal femur IM nail	77	205.5	273.9	+ 32.6%	*p* < 0.046
Phalanx ORIF	55	2.8	2.7	− 3.2%	*p* = 0.761
Humerus ORIF	35	71.8	59.7	− 16.9%	*p* = 0.693

Our survey was distributed to 44 theatre staff members (23 radiographers and 21 members of the orthopaedic department). The majority of respondents felt that communication could be improved while using intraoperative fluoroscopy. 95.2% of surgical team members and 91.3% of radiographers felt they had previously experienced communication issues during an operation using C-arm fluoroscopy. Importantly, 90.5% of the surgical team and 91.3% of radiographers had never been taught a formal method of communication or specific commands for the use of intraoperative fluoroscopy although 95.2% of surgeons and 100% of radiographers were willing to learn the new tool (Table 2).

**Table 2:** Theatre staff experiences using intraoperative C-arm fluoroscopy

**Question**	**Surgical team (21 responses)**		**Radiographers (23 responses)**	
How frequently do you use a C-arm in work?	Very often Often Sometimes Rarely	10 (47.6%) 8 (38.0%) 3 (14.4%) 0 (0.0%)	Very often Often Sometimes Rarely	5 (21.7%) 6 (26.1%) 7 (30.4%) 5 (21.7%)
Have you ever been taught a “Universal C-arm language?”	Yes 2 (9.5%) No 19 (90.5%)		Yes 2 (8.7%) No 21 (91.3%)	
Have you read the new posters in theatre displaying the new universal C-arm language?	Yes 14 (66.6%) No 7 (33.3%)		Yes 13 (56.5%) No 10 (43.5%)	
Have you ever experienced communication issues during an operation using the C-arm?	Yes 20 (95.2%) No 1 (4.8%)		Yes 21 (91.3%) No 2 (8.7%)	
Are you willing to learn a new universal C-arm language?	Yes 20 (95.2%) No 1 (4.8%)		Yes 23 (100%) No 0 (0.0%)	

**Fig. 2:**
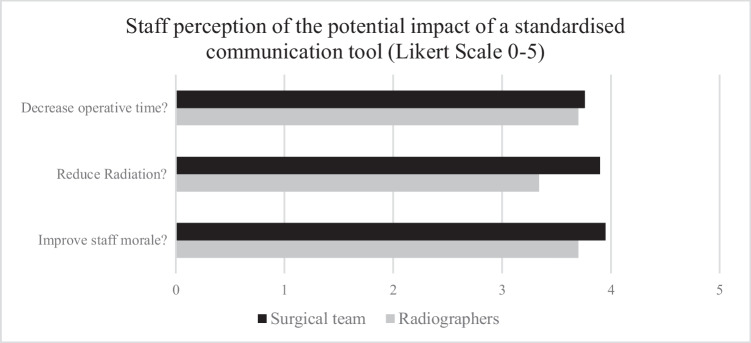
Surgical team and radiographer perception of the potential impact of the standardised communication tool. Likert scale were 0 = strongly disagree, 5 = strongly agree

We also examined staff perceptions of the potential benefits of a communication tool in theatre. The surgical team believed that it would decrease operative time (mean Likert score 3.7/5, SD 1.04), reduce radiation exposure (3.9/5, SD 1.09), and improve theatre staff morale (3.9/5, SD 1.2). The radiographers responded that they also thought the tool would reduce radiation exposure (mean Likert score 3.3/5, SD 1.3). They believed it would significantly reduce operative time (3.7/5, SD 1.06) and improve theatre staff morale (3.7/5, SD 1.11) (Fig. 2).

## Discussion

This study demonstrates that a simple educational intervention with implementation of a standardised communication tool effectively reduces patient and staff radiation exposure. While this concept has been proven effective in simulated environments, this study is the first, to our knowledge to measure its clinical efficacy.

Williams et al. conducted an experimental study of c-arm targeting and observed that consistent communication resulted in a reduction in the number of x-ray shots taken and time to a specific target. They also noted that a learning curve for commands was to be expected, but that this should ideally occur once in the lifetime of a surgeon or radiographer, rather than every time a new radiographer worked with a new surgeon. The authors recommended that a universal language is introduced at the start of surgical and radiographer training to ease this transition [5]. Similarly, Yeo et al. demonstrated that consistent commands to control the image intensifier can result in a decrease in the time required to obtain desired images and reduce radiation exposure [8].

Awareness that inadequate communication during intraoperative fluoroscopy use is a contributing factor to increased radiation exposure has gained international attention recently. A survey of 261 members of the Canadian Orthopaedic Association (COA) demonstrated that surgeons use a wide variety of terminology during procedures, including, but not limited to non-verbal gesturing, “wigwag” and “swivel”. It was highlighted that ambiguous commands, such as “Up”, which could refer to both the vertical elevation and movement towards the patient’s head, were problematic [9].

In a survey conducted by Stirton et al., surgeons and radiation technologists both believed that a standardised tool had the potential to reduce radiation exposure, operative time, and improve communication. Similar to the results of our study, 89% of surgeons and 91% of radiographers had not received formal training on how to communicate using the C-arm, although 83% of surgeons and 95% of technologists were willing to learn such a tool [6]. Burke et al. also performed a survey after implementing a standardised communication tool and found that both surgeons and radiographers agreed there was a significant improvement in perceived quality of theatre communication [6].

Occupational safety has become an increasingly important aspect of surgical practice, with radiation exposure being one of the hazards that is well acknowledged but often poorly understood by surgeons. Orthopaedic surgeons, in particular, face the highest radiation exposure to their hands, eyes, and thyroids when compared to other specialties, given their close proximity to radiation sources while operating [2]. Recent studies have also shown that female orthopaedic surgeons have a higher risk of breast cancer compared to the general US female population due to exposure to radiation [10].

Radiation dose exposure is measured in Grays (Gy), which refers to the absorbed dose or Sieverts (Sv), which refers to the effective dose. The effective dose takes into account the absorbed dose and a radiation weighting factor, which varies for different organs. In the simplest cases where the whole body is uniformly exposed to gamma and beta (electron) radiation, the radiation weighting factor and tissue weighting factor are both equal to 1. Therefore, 1 microgray (uGry) is equivalent to 1 microsievert (uSv), 0.1 milli-rem (mrem), and 0.1 milli-rad (mrad).

During orthopaedic procedures, patients and theatre staff are exposed to significant and modifiable doses of radiation. The surgeon’s hands are the body part most heavily exposed to the effects of radiation. In an in vitro study, Rampersaud et al. demonstrated that radiation exposure to the hands was 58.2 mrem/min during pedicle screw placement which was 10–12 times higher than exposure in the extremities [11]. For reference, a chest radiograph exposes the patient to approximately 25 mrem and a hip radiograph to 500 mrem. A regular C-arm exposes the patient to 1200–4000 mrem/minute. It is also worth noting that if the surgeon’s hand is within the beam, the dose is 100 times higher than the dose 15 cm from the beam where only scattered radiation affects the hand [12]. Despite this, a typical surgeon’s annual dosimetry levels are still well short of the recommended upper limits of exposure which are 5000 mrem to the body and 50,000 mrem to the hands [13–15].

Our data identified that femoral intramedullary nailing resulted in significantly more radiation exposure compared to other procedures, which has been documented previously in the literature. Sanders et al. found that 1 femoral nailing results in exposure of 6.26 min and to 100 mrem of exposure to the surgeon’s hands per operation, despite the operation time being shorter than other procedures. Fluoroscopic time was also 2.6 times longer when distal interlocking was performed, highlighting the increased radiation exposure if a long IM nail is used compared to a short IM nail. The authors concluded that the larger tissue mass around the femur resulted in more radiation scatter compared to the tibia nailing [16].

Our findings revealed that orthopaedic trainees and radiographers expressed a need for radiation safety training and emphasised the importance of incorporating a universal language tool into their training programme. Knowledge and attitudes towards radiation protection have been assessed in an Irish context previously. According to Nugent et al., Irish orthopaedic trainees showed awareness of the ALARA principles and good practices related to lead protection. However, just over half of trainees considered their training in this area to be sufficient [17]. Another study involving urology fellows and residents in the USA revealed that 53% of respondents felt that they received sufficient training in safe radiation practices, indicating that this issue is not limited to Irish trainees [18]. Example may be taken from Gendelburg et al. who demonstrated an effective training model and tangible education solution to reducing radiation exposure during mini C-arm use while reducing paediatric forearm fractures [19].

Implementing a standardised communication tool poses several challenges. The chosen terminology must be concise and applicable to all procedures involving the entire skeleton, without relying on the individual preferences of the surgeon, radiographer, or patient. Additionally, our data suggests that there is a higher percentage of radiographers who use the C-arm only “sometimes” or “rarely”, making it less likely for them to become familiar with specific terminology used by certain surgeons. There have been a number of suggested “universal languages”. Stroh et al. used a Delphi consensus method to establish their tool for C-arm communication. They proposed that achieving an ideal nomenclature was elusive but that the crucial factor for success is to establish clear and mutually agreed-upon commands for major movements and to ensure adherence to them [19].

There were a number of useful suggestions provided during our study to improve the effectiveness of the tool and streamline its implementation into other orthopaedic departments. We recommend that periodic education is provided on a C-arm commands to surgical staff and radiographers each trainee rotation. Radiographers felt strongly that one operating surgeon orders an x-ray and uses a single pre-agreed term for “x-ray”. They also felt that using approximate distances to guide the C-arm movements, e.g., push in 5 cm vs. “a little bit”, would improve accuracy (Table 3).

**Table 3:** Surgical team and radiographer attitudes/suggestions for optimising theatre communication and the introduction of a standardised C-arm communication tool

**Comments from surgical team**	**Comments from radiographers**
“The proposed system is good.”	“A single term for the command “x-ray” would be useful.”
“It will definitely reduce radiation exposure to theatre personnel.”	“No changes required to the current proposed language but surgeons should move the relevant area into the middle of the field to avoid cumbersome C-arm movements as much as possible (e.g. for wrists, fingers, ankles).”
“Will not make a huge difference but will be useful.”	“One surgical team member asking for “x-ray” is important to avoid confusion.”
“No comments: Clear and concise.”	It is not necessary to differentiate between “pushing the base vs pushing the arm” as well as “swinging the base vs swinging the arm.”
“Larger posters than A4 size would be better for theatre display.”	“Consistent use of terms for all movements is essential.”
	“Distances should be communicated in approximate measurements to improve accuracy e.g. “push in 5 cm as opposed to saying “a little bit.”
	“Would it be possible for surgeons to talk to radiographers before the case to advise regarding set-up and then what the main C-arm instructions will be?”
	“Medial and lateral could be used as instructions (Never left and right).”
	“I like using the instructions “Up and Down” to change C-arm height and “North and South” for moving proximally and distally.”
	“The image intensifier should be brought as low as possible to reduce dose exposure.”

## Limitations

While our study is limited to one orthopaedic department and its specific results may not be generalisable, we believe that its overall message is relevant to all units and may even extend to other surgical specialties that use intraoperative fluoroscopy. Of note, our data was not stratified by case complexity which would have affected the duration of radiation exposure. We did not measure patient BMI or apply a weighting for the experience of the lead operating surgeons, which may have accounted for variations in exposure during hip surgery especially. Also, the use of the laser beam was not recorded, although all C-arms used in this study were laser enabled. Furthermore, the fact that survey participants and radiation doses were being monitored could have resulted in the Hawthorne effect, which may have impacted the study’s findings. Comparing results from this study with those from other institutions or on a larger scale may help mitigate this effect.

## Conclusion

A standardised communication tool reduces staff and patient radiation exposure. The majority of surgeons and radiographers wanted to use and be educated on standard commands for the C-arm which should ease its implementation into practice. Incorporating a universal language tool into training programmes can enhance radiation safety and communication. Our study provides useful feedback from surgeons and radiographers that should help implementing a universal language into other orthopaedic departments.

## Data Availability

N/A.
